# Effect of tDCS targeting the dorsolateral prefrontal cortex on psychophysiological responses and brain oxygenation during exercise in healthy adults: a proof-of-concept randomized controlled trial

**DOI:** 10.3389/fphys.2025.1703766

**Published:** 2025-11-20

**Authors:** Daniel Gomes da Silva Machado, Mercia Jaynne Leandro de Melo, Matheus Alves Mendonça, Daniel de Medeiros Leiros, Everton Borges da Silva, Rodrigo Menezes Forti, Rickson C. Mesquita, Heloiana Faro, Edmund O. Acevedo

**Affiliations:** 1 Research Group in Neuroscience of Human Movement (NeuroMove), Department of Physical Education, Federal University of Rio Grande do Norte, Natal, Rio Grande do Norte, Brazil; 2 Institute of Physics, University of Campinas, Campinas, São Paulo, Brazil; 3 Division of Neurology, Children’s Hospital of Philadelphia, Philadelphia, PA, United States; 4 School of Computer Science, University of Birmingham, Birmingham, United Kingdom; 5 Department of Kinesiology and Health Sciences, Virginia Commonwealth University, Richmond, VA, United States

**Keywords:** transcranial direct current stimulation, exercise regulation, exercise tolerance, affective responses, perceived exertion, fNIRS

## Abstract

**Background:**

Physical inactivity is a major public health challenge, and strategies to improve exercise adherence are crucial. Affective responses play a key role in exercise behavior, and transcranial direct current stimulation (tDCS), a non-invasive technique that applies low-intensity current to the scalp, may modulate these responses. However, evidence supporting its effectiveness remains equivocal.

**Objective:**

This study aimed to evaluate the effects of tDCS targeting the dorsolateral prefrontal cortex (DLPFC) on psychophysiological responses and brain oxygenation in healthy adults.

**Methods:**

Participants first completed a maximal incremental exercise test to assess exercise performance and aerobic fitness. In two subsequent sessions, participants received either active tDCS over the DLPFC or sham stimulation for 20 min, followed by a 20-min vigorous-intensity exercise session. Heart rate (HR) and brain oxygenation (measured via functional near-infrared spectroscopy, fNIRS) were continuously monitored during exercise. Affective valence, arousal, and rating of perceived exertion (RPE) were self-reported every 5 min. Data were analyzed using Generalized Estimating Equations and Generalized Mixed Models, with significance set at p < 0.05.

**Results:**

Brain oxygenation increased after exercise compared to baseline and post-tDCS (p < 0.05). However, tDCS did not significantly alter brain oxygenation at rest (p > 0.3) or during exercise (p > 0.09). No significant effect of tDCS was observed for the affective responses (p > 0.08), arousal (p > 0.85), or RPE (leg: p > 0.90; whole-body: p > 0.28).

**Conclusion:**

DLPFC-targeted tDCS does not modulate brain oxygenation or enhance psychophysiological responses during vigorous-intensity exercise in healthy individuals. Future studies should explore exercise preference and tolerance, and the effects of tDCS on clinical populations.

## Introduction

Regular exercise improves physical fitness and is associated with health benefits, enhanced quality of life, and reduced premature mortality (Zhao et al., 2014; Imboden et al., 2018). Despite these benefits, physical inactivity remains prevalent worldwide, posing a significant public health challenge (Guthold et al., 2018; Blair, 2009). Strategies to increase physical activity and improve adherence to it are therefore essential.

Physical inactivity results from low engagement and high dropout rates (Sperandei et al., 2016; Faro et al., 2024). Emotional factors, such as prior experiences and psychophysiological responses to exercise, significantly influence adherence to physical activity (Rhodes et al., 2017; Rhodes and Kates, 2015). Among these, perceived exertion (RPE), affective responses, and arousal during exercise have been shown to be of consequential influence. Affective responses, in particular, play a central role in shaping exercise-related experiences (Ekkekakis, 2009; Ekkekakis et al., 2011; Ekkekakis, 2017). According to the Hedonic Theory of Motivation, activities eliciting pleasure are more likely to be repeated, while those causing displeasure are avoided (Kahneman et al., 1999). Systematic reviews confirm that positive affective responses during exercise predict greater physical activity behavior months later (Rhodes et al., 2017; Rhodes and Kates, 2015).

The Dual Process Theory describes decision-making as involving two systems (Ekkekakis, 2017). System 2 (rational) relies on cognitive resources to weigh the pros and cons of behaviors and predict outcomes (Ekkekakis, 2017). In contrast, System 1 (affective) operates automatically, pairing stimuli with valences (positive/negative) based on past experiences (Ekkekakis, 2017). When these systems conflict, System 1 seems to dominate System 2 (Ekkekakis, 2017). This is critical because health interventions typically target System 2, assuming that informed individuals will make rational decisions to attain their goals (e.g., living better or longer). However, behaviors such as smoking and physical inactivity persist despite awareness of their risks. Thus, modifying the affective response to an exercise session can foster positive experiences that, ultimately, may be associated with greater exercise adherence (Rhodes et al., 2017; Rhodes and Kates, 2015; Ekkekakis, 2009; Ekkekakis et al., 2011; Ekkekakis, 2017; Kahneman et al., 1999).

The brain integrates bodily signals during exercise, generating perceptions of effort, pain, arousal, and affect. The prefrontal cortex (PFC) has a central role in processing cognitive and emotional information during exercise (Robertson and Marino, 2016; McMorris et al., 2018), while the insula and pain-related regions also contribute to affective responses (Craig, 2015; Craig, 2002; Craig, 2009). Modulating activity in these regions may improve affective responses to exercise. Transcranial Direct Current Stimulation (tDCS) involves applying a low-intensity electrical current (up to 4 mA) to the scalp through two electrodes, one anodal and one cathodal. Stimulation lasts for approximately 20 min and can modulate neuronal excitability in a bipolar manner, with anodal stimulation increasing excitability and cathodal stimulation decreasing it (Kuo et al., 2013; Nitsche and Paulus, 2000). tDCS offers several advantages over other neuromodulation techniques, including lower cost, ease of application, and greater portability (Davis, 2013).

Studies have explored tDCS effects on physical performance in healthy individuals (Angius et al., 2016; Angius et al., 2015; Vitor-Costa et al., 2015; Okano et al., 2015; Barwood et al., 2016; Machado et al., 2019; Machado et al., 2021), with some demonstrating reduced RPE during exercise in athletes and active individuals (Okano et al., 2015; Etemadi et al., 2023; Angius et al., 2019). However, research on tDCS effects on affective responses is limited. A preliminary study found no effect of anodal tDCS targeting the insular cortex on affective responses or RPE in sedentary individuals (Okano et al., 2017). In contrast, tDCS applied to the left dorsolateral prefrontal cortex (DLPFC) reduced RPE, increased affective responses, and improved performance in active individuals during exhaustive exercise under hypoxia (Etemadi et al., 2023), suggesting region-dependent effects. tDCS also modulates brain activity, which can be assessed using functional near-infrared spectroscopy (fNIRS) to measure cerebral oxygenation (Herold et al., 2018). A systematic review indicates that tDCS alters brain activity at both stimulation sites and distal regions, likely due to its influence on neural networks (Patel et al., 2020).

Despite these findings, most studies have focused on the effects of tDCS on physical performance, with limited attention to the exercise experience in healthy, non-athlete individuals. In addition, few studies have examined brain activity to identify central mechanisms underlying tDCS effects. Therefore, we evaluated the effects of tDCS targeting the DLPFC on psychophysiological responses and brain oxygenation during exercise in healthy adults. We hypothesized that tDfCS applied before exercise would increase affective valence (Etemadi et al., 2023) and reduce RPE (Etemadi et al., 2023; Angius et al., 2018) during exercise and increase brain oxygenation (Patel et al., 2020).

## Methods

### Study design

This was a randomized, controlled, crossover, single-blind trial. Evaluations were conducted across three separate laboratory visits. During the first visit, participants were informed about the study’s objectives and procedures. Those who agreed to participate were screened for the inclusion criteria, and they were enrolled following informed consent. After enrollment, characterization data were collected, including anthropometric and body composition measurements, and participants completed a maximal incremental exercise test. Specific measurement and test protocols are described below.

**FIGURE 1:**
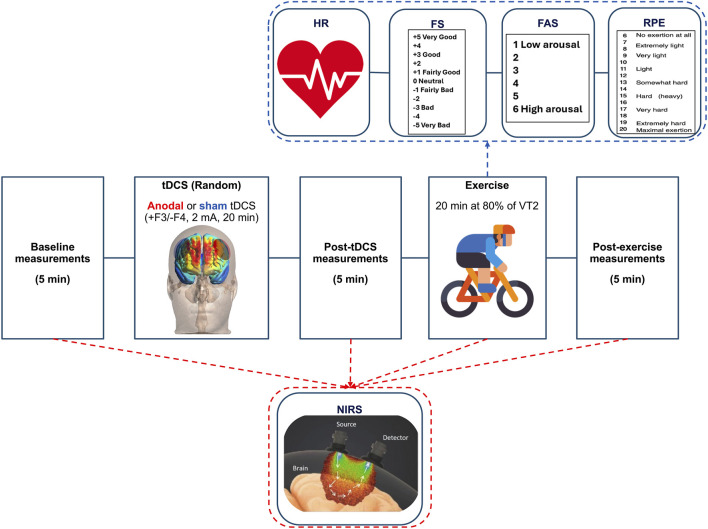
Schematics of the experimental sessions. Dashed arrows indicate the variables measured during each phase of the experimental session. HR: heart rate; FS: Feeling Scale; FAS: Felt Arousal Scale; RPE: Ratings of perceived exertion; tDCS; Transcranial Direct Current Stimulation; +F3: anodal electrode over the left dorsolateral prefrontal cortex; −F4: cathodal electrode over the right dorsolateral prefrontal cortex; VT2: second ventilatory threshold; NIRS: Near infrared spectroscopy.

In the two subsequent visits, participants performed a 20-min exercise session preceded by either 20 min of anodal tDCS (a-tDCS) over the DLPFC or sham stimulation. Stimulation was delivered in a randomized and counterbalanced order (Figure 1). The counterbalancing of the sessions was performed using the Latin Squares method (https://hci-studies.org/balanced-latin-square/). Each stimulation order was placed in an opaque envelope and randomly selected by participants. Before and after each experimental intervention, participants’ brain oxygenation was measured. During the 20-min exercise session, heart rate (HR) and brain oxygenation were continuously monitored. Participants also reported their perceived exertion (RPE), affective valence, and arousal every 5 min. The tests were administered with a minimum interval of 48 h between sessions to avoid potential residual effects from previous sessions. Participants were evaluated at the same time of day to minimize the influence of circadian variations, in a room with an average temperature of 22.0 ± 0.8 °C and relative humidity of 77 ± 9. The study protocol was approved by the Institutional Ethics Committee (CAAE: 73111923.5.0000.5537) and the study was conducted following the Declaration of Helsinki.

### Sample

The sample consisted of 12 healthy adults (7 males and 5 females). Participants’ characteristics are presented in Table 1. The number of participants was determined based on an *a priori* sample size calculation performed using G*Power 3.1.9.2 software (Universität Kiel, Kiel, Germany) with the following criteria: test = repeated-measures ANOVA (within factors); moderate effect size (f = 0.4) (Teymoori et al., 2023; Etemadi et al., 2023); α = 0.05; study power = 80%; number of groups/conditions = 2; number of measurements = 4; correlation between measurements = 0.5; non-sphericity correction = 1. According to the sample size calculation, the ideal number of participants was n = 12.

**TABLE 1:** Participants characteristics.

Variable	Female (n = 5)	Male (n = 7)	Whole sample (n = 12)
Age (years)	21.6 ± 1.5	23.1 ± 2.3	22.5 ± 2.1
Height (m)	1.62 ± 0.05	1.73 ± 0.08	1.69 ± 0.08
Weight (kg)	60.6 ± 7.8	77.6 ± 9.6	70.5 ± 12.2
BMI (kg·m^-2^)	22.9 ± 2.3	24.9 ± 3.0	24.1 ± 2.8
Body fat (%)	32.3 ± 6.6	21.8 ± 7.1	26.2 ± 8.5
Fat free mass (kg)	39.2 ± 5.0	50.3 ± 10.9	45.6 ± 10.4
Bone density (g·cm^−3^)	1.13 ± 0.04	1.25 ± 0.09	1.19 ± 0.09
Peak power (W)	136.0 ± 26.1	203.6 ± 17.3	175.4 ± 40.3
VO_2_max (mL·kg^−1^·min^−1^)	31.0 ± 5.1	31.1 ± 4.5	31.0 ± 4.5
HRmax (bpm)	187.2 ± 5.1	183.3 ± 6.9	184.9 ± 6.3
Load at VT1 (W)	72.0 ± 11.0	105.0 ± 23.5	91.3 ± 25.1
Load at VT1 (%PP)	53.4 ± 4.8	51.9 ± 11.8	52.5 ± 9.2
HR at VT1 (%HRmax)	75.3 ± 9.9	73.5 ± 6.9	74.3 ± 7.9
VO_2_ at VT1 (%VO_2_max)	55.8 ± 11.0	56.1 ± 5.4	56.0 ± 7.7
Load at VT2 (W)	128.0 ± 17.9	163.6 ± 21.4	148.8 ± 26.5
Load at VT2 (%PP)	95.0 ± 6.8	80.5 ± 10.1	86.6 ± 11.3
HR at VT2 (%HRmax)	93.7 ± 5.1	88.5 ± 5.1	90.7 ± 5.6
VO_2_ at VT2 (%VO_2_max)	81.3 ± 13.8	78.3 ± 2.8	79.6 ± 8.7
Load at 80% VT2 (W)	102.4 ± 14.3	134.3 ± 9.8	121.0 ± 19.9

BMI: body mass index; HR: heart rate; VO_2_max: maximum oxygen uptake; VT1: first ventilatory threshold; VT2: second ventilatory threshold.

The inclusion criteria were: age between 18 and 40 years, no history of neurological or psychiatric disorders, no use of medications that could interfere with the outcome variables, no history of epileptic seizures, no implanted medical devices or metal pieces in the head, no frequent or severe headaches, and no contraindications to exercise. The exclusion criteria were: voluntary withdrawal, inability to complete the exercise sessions, or impossibility to analyze the data. Participants were recruited through the distribution of a flyer containing information about the study’s objectives and procedures on social media platforms (i.e., Instagram and WhatsApp). Individuals who contacted the research team and expressed interest in participating received information about the study’s objectives and procedures. Potential participants who met the inclusion criteria and agreed to participate by signing the informed consent form were enrolled as research participants.

The data acquisition for female participants was planned so that the experimental sessions occurred during the follicular phase of the menstrual cycle (Allen et al., 2016). The determination of menstrual cycle phases was based on prospectively counting the number of days from the onset of menses (forward counting) and counting backward the number of days from the last menses (backwards counting) (Allen et al., 2016). The incremental test was performed in the last 2 days of menses, estimated based on cycle history and confirmed *post hoc*, whereas the experimental sessions were performed during the follicular phase, characterized as day 1 to day 5 after cessation of menses, confirmed by self-report.

### Initial assessment

During the first session, participants were evaluated for their eligibility to participate in the present study and completed the Physical Activity Readiness Questionnaire (PAR-Q) (Schwartz et al., 2021), which assesses their readiness for physical activity and determines whether a medical evaluation is required before starting an exercise program. Participants who provided all negative responses on the PAR-Q, meaning they had no contraindications for physical exercise and did not require medical clearance before beginning an exercise program, were included in this study.

### Anthropometric and body composition assessment

Body mass (in kilograms) and height (in meters) were measured using a digital scale with an attached stadiometer (Welmy®, W110H, Santa Bárbara d´Oeste, SP, Brazil), with a precision of 0.1 kg and 0.01 m, respectively. Body mass index (BMI) was calculated as the ratio of weight to height squared (kg/m^2^).

Additionally, dual-energy X-ray absorptiometry (DEXA) was used to assess fat-free mass and fat mass. Measurements were performed using a Lunar Prodigy device (GE Medical System, Madison, WI, USA) through whole-body scanning. Prior to the assessments, the equipment was calibrated according to the manufacturer’s recommendations. Participants were assessed wearing light clothing, without shoes, and without any metal objects or accessories on their bodies. Participants remained lying still in the supine position, with hands pronated, ankles adducted, and in plantar flexion until the measurement was completed. After the body scan, the body segments were demarcated by standardized lines generated by the software, which were adjusted if needed according to specific anatomical landmarks. DEXA measurements demonstrate excellent test-retest reproducibility (intraclass correlation coefficient, ICC ≥ 0.991) (Machado et al., 2021).

### Incremental test

The incremental test was performed on an electromagnetically braked cycle ergometer (CG04, Imbramed, Porto Alegre, RS, Brazil). We followed the protocol presented by Gaskill et al. (2001), with an initial load of 40 W for women and 50 W for men, and increments of 20 W and 25 W, respectively. Participants were required to maintain a cadence of 60–80 rpm. The cycle ergometer setup (seat height, horizontal distance of the saddle, and handlebar position) was adjusted for each participant and recorded for replication in the subsequent sessions. During the test, heart rate (HR) and respiratory variables were continuously monitored. The test was terminated upon voluntary exhaustion or if the participant could not maintain a minimum cadence of 60 rpm for >5 s. At the end of each stage, participants were asked to report their affective valence, rating of perceived exertion (RPE) and arousal. HR and respiratory variables were continuously measured throughout the test.

### Transcranial direct current stimulation

**FIGURE 2:**
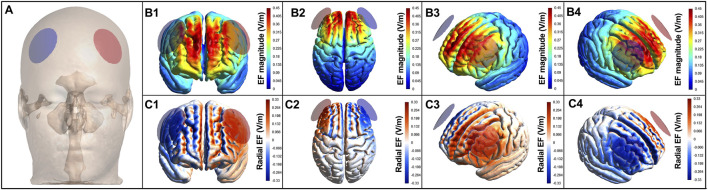
Electric field induced by tDCS in the brain. Electric field induced by tDCS in brain areas for the montage targeting the left dorsolateral prefrontal cortex. Anodal electrodes (red circles) and cathodal electrodes (blue circles) placed on the scalp **(A)**. **(B)** are color-coded according to the intensity of the electric field, with hot colors (e.g., red) representing stronger electric fields and cold colors (e.g., blue) representing weaker electric fields. **(C)** are color-coded according to the direction of the electric field, with warm colors representing inward currents and cold colors representing outward currents.

We applied tDCS using two circular electrodes (diameter of 4.1 cm) enwrapped in a sponge (diameter of 6.0 cm; NKL, Brusque, SC, Brazil) embedded with approximately 10 mL of saline solution. Electrodes were attached to the participant’s head using a neoprene cap following the 10–20 EEG positioning system (NKL, Brusque, SC, Brazil). The electrical current was applied using an automated tDCS device (MicroStim Focus Research, NKL, Brusque, SC, Brazil) with a ramp up and down of 30 s, with an intensity of 2 mA for 20 min. The stimulation started with impedance below 20 kOhm. The anode electrode was placed over F3 and the cathode electrode over F4, according to the 10–20 EEG system (Figure 2A). For the sham condition, we applied a 2-mA current for 30 s, with a 30-s ramp-up and ramp-down period, after which the current remained turned off for the remainder of the session. This sham tDCS procedure has been shown to effectively blind participants (Teymoori et al., 2023; Etemadi et al., 2023; Gandiga et al., 2006; Moreira et al., 2021). Participants were not informed of the tDCS condition they were receiving. The device was kept behind the participants, and the screen displayed the same information for both active and sham sessions; only the codes for actual and sham stimulations were different. Moreover, participants were not informed about the sham condition (Teymoori et al., 2023; Etemadi et al., 2023) to prevent expectation effects (Rabipour et al., 2018; Turi et al., 2018), even though such placebo effects of tDCS on motor performance have been recently questioned (Borges da Silva et al., 2025). After participants had concluded their involvement in the study were they fully debriefed about the sham session, the study hypothesis, and their respective performance.

### tDCS-induced electric field simulation

The brain current flow during tDCS was calculated using a finite element model following the standard pipeline in SimNIBS 4.0.0 (Thielscher et al., 2015). The magnetic resonance imaging (MRI) MNI 152 head model available in the software was used. MRI data were segmented into surfaces corresponding to the white matter (WM), gray matter (GM), cerebrospinal fluid (CSF), skull, and skin. The electrical conductivities of each segment were determined according to values previously established as follows: WM = 0.126 S/m (S/m), GM = 0.275 S/m, CSF = 1.654 S/m, bone = 0.010 S/m, and skin/scalp = 0.465 S/m (Opitz et al., 2015), rubber electrode = 29.4 S/m, and saline-soaked sponges = 1.000 S/m. All information concerning the respective tDCS montages was entered into the software: current intensity = 2 mA; electrode position (+F3/−F4); electrode (4.1 cm) and sponge sizes (6.0 cm); electrode thickness = 1 mm; sponge thickness = 5 mm. The results of the simulations are presented in Figure 2, in terms of the electric field magnitude and radial electric field (normal to the cortical surface), both of which are the most important variables for tDCS to exert its neuromodulatory effects (Rahman et al., 2013). As can be seen in Figure 2, tDCS was able to reach our nominal target with enough electric field magnitude (Figure 2B) and with inward current (Figure 2C), which is expected to induce a neuromodulatory effect (Neri et al., 2020). The DLPFC tDCS montage also reached other prefrontal areas between the electrodes, such as the bilateral ventromedial and ventrolateral PFC, and right DLPFC (Figure 2).

### tDCS-induced sensations and blinding assessment

To assess the effectiveness of participant blinding, participants completed a questionnaire after each experimental session that listed the sensations and their intensity levels experienced during the stimulation (Fertonani et al., 2015). This questionnaire has been used in previous studies involving tDCS and exercise (Teymoori et al., 2023; Etemadi et al., 2023; Machado et al., 2021). The questionnaire included sensations such as itching, pain, burning, warmth/heat, tingling, metallic/iron taste, fatigue, and other sensations (open-ended questions). The intensity of each sensation was rated on a scale of: none (0), mild (1), moderate (2), considerable (3), and strong (4). Participants also indicated whether these sensations affected their ability to perform the exercise (0 = not at all; 1 = slightly; 2 = considerably; 3 = much; 4 = very much); when the discomfort began (1 = at the beginning; 2 = around the middle; 3 = toward the end); and when it stopped (1 = stopped quickly; 2 = stopped in the middle; 3 = stopped at the end). The overall “discomfort” generated by tDCS was calculated as the sum of the intensity scores for all sensations, ranging from 0 (no discomfort) to 28 (maximum discomfort).

### Fixed load sessions

In the experimental sessions, after tDCS application, participants performed a fixed-load exercise session on a cycle ergometer (CG04, Imbramed, Porto Alegre, RS, Brazil). The load for the fixed-load sessions was determined as 80% of the individually determined load corresponding to the second ventilatory threshold (VT2). The sessions began with a 3-min warm-up, starting at 40 W for women and 50 W for men. The load was then increased in two 1-min steps until the target load was reached at 3 min. The target load was maintained for 20 min. Cycling cadence was kept between 60 and 80 rpm. During the exercise, HR and respiratory variables were continuously monitored. Every 5 min, participants reported their RPE, arousal, and affective responses.

### Rating of perceived exertion, affective valence, and arousal

RPE was measured using the 15-point Borg scale (6–20) (Borg, 1998). The scale assesses the intensity of physical effort or work, and instructions were provided in a standardized manner during the familiarization session and before each test session (Pandolf, 1982; Noble et al., 1983). Affective valence was assessed using the Feeling Scale, which is an 11-point bipolar scale ranging from −5 (“very bad”) to +5 (“very good”), with verbal descriptors placed at all odd numbers and zero (“neutral”) (Hardy and Rejeski, 1989). Arousal state was assessed using the Felt Arousal Scale, which consists of a scale ranging from 1 (“low arousal”) to 6 (“high arousal”) (Etemadi et al., 2023; Svebak and Murgatroyd, 1985; Krinski et al., 2017; Farias-Junior et al., 2023). In the first session, participants first underwent a memory familiarization in which each scale and its specific purpose were carefully explained, with examples provided for lower and higher values. Their understanding was verified before proceeding. Subsequently, the maximal incremental test was used as a practical familiarization, allowing participants to associate different exercise intensities with specific values on each scale. Psychophysiological responses were reported every 5 min during the experimental sessions. The scales were presented to participants in random order to avoid automatic responses.

### Respiratory measures

Respiratory measures were collected using a computerized gas analyzer with a breath-by-breath analysis system (Quark CPET, Cosmed, Rome, Italy) during the maximal incremental test and fixed load sessions. The equipment was calibrated before each test according to the manufacturer’s recommendations. A 20-s average was applied to the respiratory variables. The ventilatory thresholds were identified using a combination of measures following the approach suggested by Binder et al. (Binder et al., 2008). The first ventilatory threshold (VT1) was determined as: 1. the first breakpoint in ventilation (VE), 2. the point where the respiratory gas exchange ratio (RER) versus workload (WL) curve changes from flat or rising slowly to a more positive slope, 3. the first curvilinear increase in VCO2, 4. a break in the VE/VO2 slope, and 5. an increase in PetO2 after reaching a minimum. The second ventilatory threshold (VT2) was identified as: 1. the second breakpoint in VE, 2. a reduction in partial pressure of end-tidal CO2 (PetCO2) after reaching maximal intensity, 3. the point where the VE/VCO2 ratio reached its lowest value and began to increase, and 4. a deflection from linearity in the heart rate versus WL relationship. The VTs were independently identified by two individuals, and in cases of discrepancy, a third researcher was consulted. The highest value achieved over a 20-s interval during the incremental test was considered the maximal oxygen uptake (VO_2_max).

### Heart rate

HR was assessed using a digital heart rate monitor (H10, Polar Electro®, Kempele, Finland). The monitor’s electrode with transmitter was positioned on the participants’ chest (at the level of the sternum) after applying water to facilitate signal transduction. Data were recorded and transmitted continuously from the electrode to a smartphone app.

### Brain oxygenation

Brain oxygenation was assessed by analyzing cerebral oxygenation using functional near-infrared spectroscopy (fNIRS). A frequency-domain multichannel fNIRS device (Imagent, ISS, Champaign, IL, USA) was used to measure the absolute concentrations of oxyhemoglobin (HbO_2_) and deoxyhemoglobin (Hb). The device consisted of two sensors, each equipped with four pairs of light sources emitting wavelengths of 690 nm and 830 nm. The light sources were positioned at distances of 2, 2.5, 3, and 3.5 cm from the detector. The sensors were placed on the participant’s forehead, covering approximately Brodmann’s area 10 (frontopolar cortex), with the measurement area centered under Fp1 and Fp2 according to the EEG 10–20 system. Due to technical limitations of our NIRS device, it was not possible to measure the oxygenation of the DLPFC because the hair in that area would have made the measurement impossible for our NIRS sensor setup. However, the frontopolar cortex is a key brain region for processing exercise-related perceptions and regulating exercise. During physical activity, it integrates interoceptive and limbic signals to evaluate choices and encode outcome expectations, which are critical for goal-directed behavior (Robertson and Marino, 2016; McMorris et al., 2018). The frontopolar cortex then projects to the lateral prefrontal cortex, including the DLPFC and ventrolateral prefrontal cortex, which is believed to decide whether to continue or stop the activity (Robertson and Marino, 2016; McMorris et al., 2018). Furthermore, the frontopolar cortex is a primary target of transcranial direct current stimulation (tDCS) using the +F3/−F4 montage. Simulation studies indicate that this montage generates a stronger electric field in the frontopolar cortex than in the DLPFC itself (Soleimani et al., 2023).

The NIRS sensors were placed at the beginning of the assessment to obtain baseline measurements. They were removed for tDCS application and then repositioned for post-intervention measurements. The target measurement areas were marked with a skin marker to minimize sensor placement/positioning variations and reduce the time between the end of tDCS and NIRS measurement. Near-infrared light was modulated at 110 MHz and transmitted through optical fibers with a diameter of 400 µm to the tissue, then returned to the detector via optical fibers with a diameter of 3 mm. For each collection cycle, eight data acquisitions were performed and averaged to produce estimates of AC intensity, DC intensity, and phase delay (PhS) for each source-detector pair, resulting in a sampling frequency of 31.25 Hz. These values were combined to estimate the absolute optical properties using a multi-distance semi-infinite model (Fantini et al., 1994); HbO2 and Hb concentrations were calculated from the absorption coefficient using the modified Beer-Lambert law through a custom MATLAB script.

### Statistical analysis

The Shapiro-Wilk test and histogram inspection were used to verify the normality of the data distribution. The data were expressed as mean and standard deviation, or median and interquartile range, as appropriate. We used a Wilcoxon test to compare the sensations induced by tDCS. A constant of ten units was added to negative data (affective responses). We used Generalized Estimating Equations (GEE) for Gaussian distribution data (brain oxygenation), and a generalized mixed model (GMM) with a gamma distribution, an identity link function, and an unstructured correlation matrix to analyze the repeated measures for non-normal data (physiological and psychophysiological responses). Individual variability was incorporated as a non-correlated random factor to account for the analysis. The quality of the model was assessed based on the lower value of the Akaike information criterion (AIC) and the distribution of residuals. Main effects and interactions were analyzed for each dependent variable. The Bonferroni-corrected *post hoc* test was used to identify specific differences. We calculated the effect size for two paired overall means for each experimental condition (tDCS vs. sham) using Hedges’ g, which is more appropriate for small sample sizes, with a 95% confidence interval (95% CI) (Lakens, 2013). A significance level of p < 0.05 was adopted. All analyses were performed using Jamovi 2.3.26.

## Results

### tDCS-induced sensations and blinding

All participants received the experimental conditions according to the randomization. There were no serious side or adverse effects reported. The most commonly reported sensations were itching and pinching on the head, which began at the start of stimulation and either stopped at the beginning or middle of the stimulation (Table 2). No significant difference was found between conditions for tDCS-induced sensations, start or end of those sensations (p > 0.2 for all comparisons). Hence, considering the similar tDCS-induced sensations, it can be assumed that the study blinding protocol was effective.

**TABLE 2:** tDCS-induced sensations and the general sensation index (discomfort) reported by participants (n = 12).

Sensations	DLPFC a-tDCS			Sham tDCS			*W*	*p*
	Mean ± SD	Median (IQR)	n (%)	Mean ± SD	Median (IQR)	n (%)		
Itchiness	2.4 ± 1.2	3 (1.8–3)	11 (91.7)	1.9 ± 1.4	1.5 (1–2.5)	11 (91.7)	27.0	0.22
Pain	0.7 ± 1.2	0 (0–1)	4 (33.3)	0.5 ± 1.0	0 (0–0.2)	3 (25)	9.00	0.78
Burning	1.1 ± 1.2	1 (0–2)	7 (58.3)	0.7 ± 1.1	0 (0–1.3)	4 (33.3)	11.5	0.34
Warmth/Heat	0.4 ± 0.5	0 (0–1)	5 (41.7)	0.4 ± 0.7	0 (0–1)	4 (33.3)	5.00	1.00
Pinching	1.8 ± 1.1	1.5 (1–2.3)	11 (91.7)	1.9 ± 1.4	2 (1–3)	10 (83.3)	15.5	0.78
Iron taste	0.1 ± 0.3	0 (0–0)	1 (8.3)	0.0 ± 0.0	0 (0–0)	0 (0)	1.0	1.00
Fatigue	0.3 ± 0.9	0 (0–0)	2 (16.7)	0.4 ± 1.0	0 (0–0)	2 (16.7)	1.0	1.00
Other	0.1 ± 0.3	0 (0–0)	1 (8.3)	0.1 ± 0.3	0 (0–0)	1 (8.3)	0.00	N/A
Discomfort	6.8 ± 4.0	6 (3.8–11)	-	5.9 ± 5.2	4.5 (2.5–7.5)	-	39.5	0.59
Start	1.2 ± 0.6	1 (1–1)	-	1.0 ± 0.0	1 (1–1)	-	1.00	1.00
End	1.9 ± 0.9	2 (1–3)	-	1.6 ± 0.7	1.5 (1–2)	-	30.0	0.39

tDCS, transcranial direct current stimulation; DLPFC, dorsolateral prefrontal cortex; mean ± standard deviation; median (interquartile range); n (%) = indicates the number and percentage of participants who experienced a particular sensation.

### Brain oxygenation at rest

**FIGURE 3:**
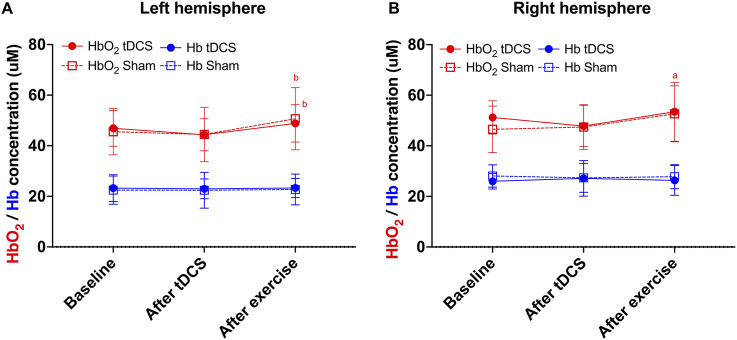
Oxygenated hemoglobin (in red) and deoxygenated hemoglobin (in blue) at baseline, after Transcranial Direct Current Stimulation (tDCS), and after exercise for the left **(A)** and right **(B)** frontopolar cortex. ^a^ = significantly different from baseline within condition (p = 0.033); b = significantly different from “after tDCS” within condition (p ≤ 0.01 for all comparisons).

Figure 3 presents the results of the brain oxygenation measured during rest at baseline, after tDCS, and after exercise cessation.

#### Left prefrontal cortex

Regarding oxyhemoglobin, a statistically significant effect of time was found (χ^2^ (2) = 13.96; p < 0.001), but no significant effect of condition (χ^2^ (1) = 0.04; p = 0.85) or interaction (χ^2^ (2) = 0.05; p = 0.97) was identified. The *post hoc* analysis indicated that the measurement taken after exercise was significantly higher than after the tDCS in both tDCS (p = 0.013) and sham conditions (p = 0.005). No other significant differences were found (Figure 3A).

Regarding deoxyhemoglobin (Figure 3A), no significant effect of condition (χ^2^ (1) = 9.09; p = 0.99), time (χ^2^ (2) = 0.03; p = 0.99) or interaction (χ^2^ (2) = 2.19; p = 0.34) was found.

#### Right prefrontal cortex

Regarding oxyhemoglobin, a statistically significant effect of condition (χ^2^ (1) = 7.92; p = 0.005) and time (χ^2^ (2) = 15.08; p < 0.001) was found, with no significant effect of the interaction (χ^2^ (2) = 1.25; p = 0.54). The *post hoc* analysis indicated that the measurement taken after exercise was significantly higher than the baseline measurement in the sham condition (p = 0.033). No other significant difference was found (Figure 3B).

Regarding deoxyhemoglobin (Figure 3B), no significant effect of condition (χ^2^ (1) = 0.75; p = 0.39), time (χ^2^ (2) = 0.08; p = 0.96) or interaction (χ^2^ (2) = 1.79; p = 0.41) was found.

### Cardiorespiratory responses during exercise

**FIGURE 4:**
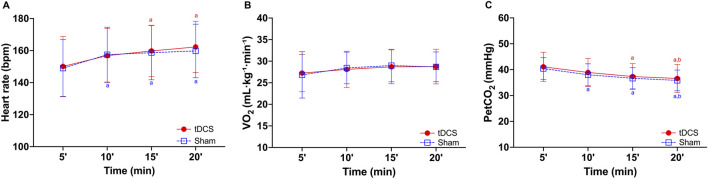
Heart rate **(A)**, oxygen uptake **(B,C)** partial pressure of end-tidal carbon dioxide (PetCO_2_) during 20 minutes of cycling exercise after receiving Transcranial Direct Current Stimulation (tDCS) or sham. ^a^ = significantly different from 5 min within condition (p ≤ 0.015 for all comparisons); ^b^ = significantly different from 10 min within condition (p ≤ 0.047 for all comparisons).

Figure 4 presents the results of the physiological assessment during exercise. The GEE revealed a significant main effect of time (F_(3, 77)_ = 22.185, p < 0.001) for HR, with no significant effect of tDCS condition (F_(1, 77)_ = 0.91, p = 0.343) or Condition × Time interaction (F_(3, 77)_ = 0.347, p = 0.791). The *post hoc* test indicated that HR was higher at 10 min (p = 0.005) in the sham condition and at 15 and 20 min in both conditions compared to 5 min (p < 0.01 for all comparisons; Figure 4A).

Similarly, a significant main effect of time (F_(3, 77)_ = 4.844, p = 0.004) was found for VO_2_, with no significant effect of tDCS condition (F_(1, 77)_ = 0.01, p = 0.919) or Condition × Time interaction (F_(3, 77)_ = 0.220, p = 0.882). The *post hoc* test indicated no significant difference (Figure 4B). Finally, a significant main effect of time was found for PetCO_2_ (χ^2^ (3) = 95.5; p < 0.001), with no significant effect of tDCS condition (χ^2^ (1) = 1.79, p = 0.181) or Condition × Time interaction (χ^2^ (3) = 0.07, p = 0.995). Post hoc tests indicated that PetCO_2_ decreased over time, with lower values at 15 and 20 min compared to 5 min in the tDCS condition, and at 10, 15, and 20 min compared to 5 min in the sham condition (all p ≤ 0.015; Figure 4C). At 20 min, PetCO_2_ was also lower than at 10 min in both conditions (all p ≤ 0.047). No significant differences were found between the two conditions.

### Psychophysiological responses

**FIGURE 5:**
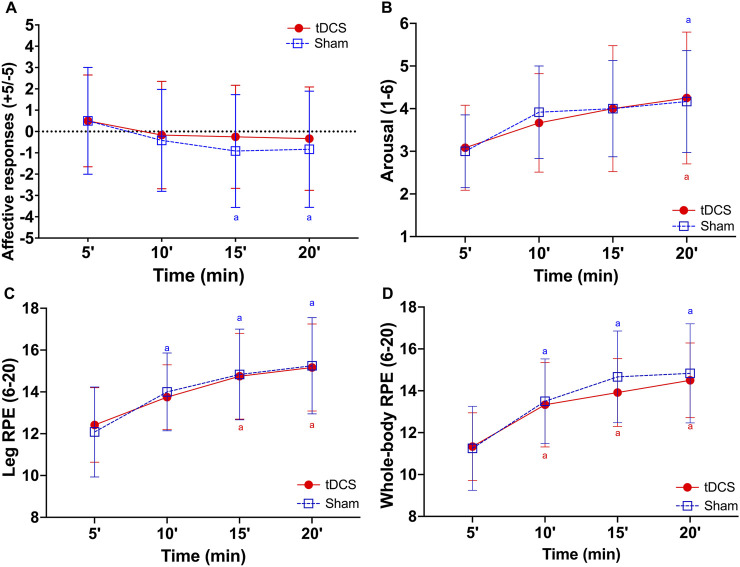
Affective responses **(A)**, arousal **(B)**, leg **(C)**, and whole-body **(D)** ratings of perceived exertion (RPE) during 20 min of cycling exercise after receiving Transcranial Direct Current Stimulation (tDCS) or sham. ^a^ = significantly different from 5 min within condition (p ≤ 0.04 for all comparisons).

Figure 5 presents the results of the psychophysiological assessment during exercise. The GEE revealed a significant main effect of time (F_(3, 77)_ = 6.50, p < 0.001) in affective responses, but not for tDCS condition (F_(1, 77)_ = 3.11, p = 0.082) or Condition × Time interaction (F_(3, 77)_ = 0.53, p = 0.665). The *post hoc* test indicated that the affective responses were lower at 15 (p = 0.02) and 20 min (p = 0.04) compared to 5 min in sham condition (Figure 5A).

Similarly, a significant main effect of time (F_(3, 77)_ = 10.664, p < 0.001) was found for arousal, with no effect of tDCS condition (F_(1, 77)_ = 0.018, p = 0.894) or Condition × Time interaction (F_(3, 77)_ = 0.257, p = 0.856). The *post hoc* test indicated that arousal was higher at 20 min (p = 0.01 for all comparisons) in both conditions compared to 5 min (Figure 5B).

A significant main effect of time (F_(3, 77)_ = 20.761, p < 0.001) was found for leg RPE, but not for tDCS condition (F_(1, 77)_ = 0.005, p = 0.942) or Condition × Time interaction (F_(3, 77)_ = 0.187, p = 0.905). The *post hoc* test indicated that leg RPE was higher at 10 min in sham (p = 0.04) condition and at 15 (p < 0.001 for all comparisons) and 20 min (p < 0.01 for all comparisons) in both conditions compared to 5 min (Figure 5C).

Likewise, a significant main effect of time (F_(3, 77)_ = 30.998, p < 0.001) was found for whole-body RPE, with no effect of tDCS condition (F_(1, 77)_ = 1.156, p = 0.286) or Condition × Time interaction (F_(3, 77)_ = 0.417, p = 0.741). The *post hoc* test indicated that whole-body RPE was higher at 10, 15, and 20 min (p < 0.01 for all comparisons) in both conditions compared to 5 min (Figure 5D).

### Brain oxygenation during exercise

**FIGURE 6:**
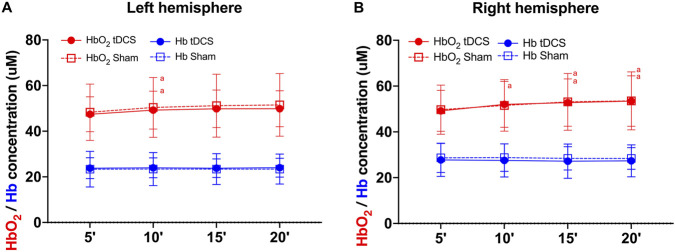
Oxygenated hemoglobin (in red) and deoxygenated hemoglobin (in blue) during 20 minutes of cycling exercise for the left **(A)** and right **(B)** frontopolar cortex after receiving Transcranial Direct Current Stimulation (tDCS) or sham. ^a^ = significantly different from 5 min within condition (p ≤ 0.049 for all comparisons).

Figure 6 presents the results of brain oxygenation measurements taken during exercise.

#### Left prefrontal cortex

Regarding oxyhemoglobin (Figure 6A), a statistically significant effect of time was found (χ^2^ (3) = 16.15; p = 0.001), but no significant effect of condition (χ^2^ (1) = 1.24; p = 0.27) or interaction (χ^2^ (3) = 0.93; p = 0.82) was identified. The *post hoc* analysis indicated that the oxyhemoglobin was significantly higher at 10 min compared to the first 5 min in both anodal (p = 0.049) and sham (p = 0.02) conditions. No other significant differences were found.

Regarding deoxyhemoglobin (Figure 6A), no significant effect of condition (χ^2^ (1) = 0.11; p = 0.75), time (χ^2^ (3) = 1.48; p = 0.69) or interaction (χ^2^ (3) = 5.23; p = 0.16) was found.

#### Right prefrontal cortex

Regarding oxyhemoglobin (Figure 6B), a statistically significant effect of time was found (χ^2^ (3) = 20.79; p < 0.001), but no significant effect of condition (χ^2^ (1) = 0.003; p = 0.96) or interaction (χ^2^ (3) = 2.32; p = 0.51) was identified. The *post hoc* analysis indicated that the oxyhemoglobin was significantly higher at all timepoints compared to the first 5 min in both anodal (p ≤ 0.001 for all comparisons) and sham (p ≤ 0.04 for all comparisons) conditions. No other significant differences were found.

Regarding deoxyhemoglobin (Figure 6B), no significant effect of condition (χ^2^ (1) = 0.03; p = 0.86), time (χ^2^ (3) = 1.26; p = 0.74), or interaction (χ^2^ (3) = 6.59; p = 0.09) was found.

The overall means and standard deviations, and effect size for paired comparisons between experimental conditions are presented in Table 3.

**TABLE 3:** Overall means and standard deviations, effect sizes, and 95% confidence intervals of the study outcome variables.

Variables	tDCS	Sham	Hedge’s g	95% confidence intervals	
				Lower limit	Upper limit
HbO_2_ at rest (uM) – LH	46.9 ± 7.1	45.5 ± 9.2	−0.17	−0.97	0.63
HHb at rest (uM) – LH	23.3 ± 5.3	22.4 ± 5.6	−0.17	−0.97	0.64
HbO_2_ at rest (uM) – RH	51.2 ± 6.6	46.5 ± 9.2	−0.59	−1.40	0.23
HHb at rest (uM) – RH	26.0 ± 3.0	28.1 ± 4.4	0.56	−0.26	1.37
HbO_2_ after tDCS (uM) – LH	44.3 ± 6.3	44.5 ± 10.8	0.02	−0.78	0.82
HHb after tDCS (uM) – LH	23.0 ± 3.9	22.4 ± 7.1	0.11	−0.91	0.70
HbO_2_ after tDCS (uM) – RH	47.8 ± 8.2	47.4 ± 8.9	−0.05	−0.85	0.75
HHb after tDCS (uM) – RH	27.1 ± 7.0	27.3 ± 5.7	0.03	−0.77	0.83
HbO_2_ after exercise (uM) – LH	48.8 ± 7.4	50.7 ± 12.3	0.19	−0.61	0.99
HHb after exercise (uM) – LH	23.3 ± 3.8	22.7 ± 6.1	−0.12	−0.92	0.68
HbO_2_ after exercise (uM) – RH	53.5 ± 11.6	52.6 ± 11.1	0.01	−0.79	0.81
HHb after exercise (uM) – RH	26.3 ± 5.9	27.8 ± 4.7	0.28	−0.52	1.09
Heart rate (bpm)	157.2 ± 17.0	156.2 ± 17.1	−0.06	−0.86	0.74
Oxygen uptake (mL·kg^−1^·min^−1^)	28.2 ± 4.0	28.2 ± 4.1	0.01	−0.79	0.81
Leg RPE (6–20)	14.0 ± 2.1	14.0 ± 2.4	0.01	−0.79	0.81
Whole-body RPE (6–20)	13.3 ± 2.1	13.6 ± 2.5	0.13	−0.68	0.93
Arousal (1–6)	3.8 ± 1.3	3.8 ± 1.1	0.02	−0.78	0.82
Affective responses (+5/−5)	−0.1 ± 2.3	−0.4 ± 2.6	−0.15	−0.95	0.65
HbO_2_ during exercise (uM) – LH	49.1 ± 7.8	50.4 ± 12.9	0.12	−0.68	0.92
HHb during exercise (uM) – LH	23.9 ± 4.1	23.4 ± 6.9	−0.09	−0.89	0.71
HbO_2_ during exercise (uM) – RH	51.9 ± 9.9	52.0 ± 11.5	0.01	−0.79	0.81
HHb during exercise (uM) – RH	27.5 ± 7.0	28.6 ± 5.4	0.18	−0.63	0.98

HbO_2_: oxyhemoglobin; HHb: deoxyhemoglobin; LH: left hemisphere; RH: right hemisphere; RPE: ratings of perceived exertion; tDCS: transcranial direct current stimulation.

## Discussion

The main results of the present study were that a single session of anodal tDCS targeting the left DLPFC did not change the affective response, arousal, or RPE during exercise. Additionally, tDCS did not affect brain oxygenation at rest or during exercise. To the best of our knowledge, this was the first study to evaluate the effect of DLPFC-tDCS on psychophysiological responses during a fixed intensity exercise in healthy individuals and brain oxygenation responses at rest and during exercise.

The lack of an effect of tDCS on psychophysiological responses during exercise was surprising considering that previous studies have shown a significant effect on those responses. For example, Etemadi et al. (2023) found that tDCS targeting DLPFC, but not primary motor cortex (M1), resulted in higher affective responses and arousal and reduced RPE during a time to exhaustion (TTE) test in hypoxia in endurance-trained males. Similarly, Teymoori et al. (2023) reported increased affective responses and lower RPE during repeated all-out cycling sprints in physically active healthy males after DLPFC- but not M1-tDCS. Considering that tDCS improved psychophysiological responses in samples of physically active and well-trained individuals, we hypothesized that tDCS could improve those variables in aerobically unfit individuals, but this was not supported by our results. However, the present results also align with other studies that did not find significant effects of tDCS targeting different brain regions on psychophysiological responses. Machado et al. (2021) reported no significant effect of conventional or high-definition M1-tDCS on RPE during TTE in endurance-trained individuals, which was similar to Etemadi et al. (2023) and Teymoori et al. (2023), who found no effect of M1-tDCS. Okano et al. (2017) also found no effect of temporal cortex tDCS on affective responses and RPE in sedentary males during a 30-min vigorous intensity cycling. The discrepancy between studies may be partially explained by differences in baseline neurophysiological function (Li et al., 2015). Previous research suggests that the effects of neuromodulation techniques like tDCS are state-dependent, influenced by factors such as whether a subject is awake, asleep, or in a particular affective or cognitive state (Bergmann, 2018; Li et al., 2019; Schutter et al., 2023). In this context, more trained individuals exhibit distinct profiles of brain activity, structure, and cognitive performance compared to their less trained peers (Jacobson and Matthaeus, 2014; Nakata et al., 2010; Faro et al., 2020; Ren et al., 2025), a finding that aligns with the neural efficiency theory (Li and Smith, 2021; Ludyga et al., 2015; Del et al., 2008).

We also evaluated the possible effect of tDCS on brain oxygenation and found no change in oxy- and deoxy-hemoglobin due to stimulation, neither at rest nor during exercise. fNIRS is an indirect measure of brain activity based on the neurovascular coupling, a multidimensional process orchestrated by the neurovascular unit, involving vasoactive mediators released from multiple cell types (including neurons, astrocytes, and endothelial cells) that engage distinct signaling pathways across the entire cerebrovascular network to modulate blood flow delivery to support neural activity (Herold et al., 2018; Iadecola, 2017). Hence, considering that tDCS may influence neuronal excitability and spontaneous firing rate, although it does not directly make neurons fire (Pelletier and Cicchetti, 2015), it was hypothesized that tDCS could influence brain oxygenation. Recent evidence has also suggested that tDCS might have a direct effect on the vasculature itself that could cause vasodilation independently of changes in neuronal activity (Shin et al., 2020; Bahr-Hosseini and Bikson, 2021). Thus, an increase in blood flow and changes in brain oxygenation could also occur via this alternative mechanism. However, our results did not show any significant changes in brain oxygenation on the PFC following anodal tDCS. In addition, considering that anodal tDCS increases the likelihood of neurons firing and impacts ongoing activities (Pelletier and Cicchetti, 2015), a synergistic effect between tDCS and exercise could be expected, resulting in increased oxyhemoglobin during exercise compared to sham. However, there was also no difference in brain oxygenation during exercise at any time point. The only significant difference was an increase in oxyhemoglobin after exercise cessation compared to baseline and post tDCS, which is a known effect of exercise of intensities ranging from light to the respiratory compensation point (De Wachter et al., 2021).

Despite not supporting the initial hypothesis, our results align with previous literature concerning the effect of tDCS on brain oxygenation at rest (Radel et al., 2017; Yan et al., 2015). For example, Radel et al. (Radel et al., 2017) found no difference in prefrontal cortex brain oxygenation measurements at rest between baseline and post-tDCS targeting either the PFC or M1. The studies that did find significant changes in brain oxygenation with tDCS used, in general, lower tDCS intensity (i.e., 1 mA) and/or shorter duration (i.e., ≤10 min), with a handful testing commonly used tDCS parameters (i.e., 2 mA for 20 min) (Patel et al., 2020). Nevertheless, a systematic review by Patel et al. (2020), reported an increase in brain oxygenation at the site of stimulation and in regions distal to the stimulation target. The different results among studies might be explained by the differences in the nominal brain targets for tDCS and the region of interest for fNIRS measurements, and also the lack of a sham/control group in some studies (Patel et al., 2020). In terms of measurements of brain oxygenation during exercise, while studies evaluating tDCS applied during exercise showed a general decrease in cortical activation (Radel et al., 2017; Patel et al., 2020), the two studies that assessed brain oxygenation offline (i.e., after tDCS) found no change (Muthalib et al., 2013; Besson et al., 2019). Differences in results among the present and previous studies may be explained by differences not only in tDCS protocol (i.e., intensity, duration, nominal target) but also in the exercise type and protocol (i.e., single joint vs. whole-body exercise, isometric time to task failure or finger taping vs. cycling).

The possible explanation for the lack of a significant influence of tDCS on the outcome measure might be related to the absence of a long-term tDCS-induced neuromodulatory effect. If we consider that brain oxygenation represents an objective measure of the neuromodulation effect secondary to tDCS (Patel et al., 2020) or as a measure of a potential direct effect of tDCS on the vasculature (Shin et al., 2020; Bahr-Hosseini and Bikson, 2021), the fact that there was no significant change from baseline to post-tDCS indicates that neither existed. Therefore, it would not be expected to be a significant change in the psychophysiological responses since there was no neuromodulation in the first place. The discussion on the factors that might influence the neuromodulatory capability of tDCS is a matter of growing interest in the brain stimulation field and involves intra- and interindividual variability, anatomical variations, baseline level of function, local circuit, emotional state, among others (Li et al., 2015). In addition, sex differences may influence tDCS-induced electrical fields in the brain and the effects of neuromodulation techniques on the outcomes (Russell et al., 2017; Rudroff et al., 2020; Kuo et al., 2006), suggesting sex as a moderating variable. Finally, neurophysiological studies have shown that only about 50% of individuals seem to respond to M1-targeted tDCS (Wiethoff et al., 2014; López-Alonso et al., 2015), and among responders, the classical polarity-dependent effect of tDCS (i.e., “anodal-excite/cathodal-inhibit”) is not always present (Wiethoff et al., 2014). Therefore, future studies are warranted to investigate possible predictors of neuromodulation and changes in the outcome measures induced by tDCS to help in defining those who might benefit from tDCS.

### Limitations and future directions

The results of the present study must be interpreted considering its limitations, despite our attempt to control as much as possible for any intervening variables. The relatively small sample size may limit statistical power. However, as a proof of concept, this study may lay the foundation for larger trials. In addition, if the effect of an intervention requires a huge sample size to be detected, its clinical or practical significance might be negligible. Additionally, participants’ preferences for and tolerances of exercise intensity were not considered. Future studies should consider evaluating larger sample sizes, other populations, especially individuals with mental health conditions, and examining preferences and tolerances for exercise intensity.

On the other hand, the strengths of the present manuscript include its robust methodological design, with exercise intensity individually defined based on gold-standard measures for physiological monitoring during exercise. Additionally, the study monitored respiratory and cardiovascular responses during exercise, as well as brain oxygenation, both at rest and during exercise, which enhances rigor and understanding compared to the previous literature.

## Conclusion

The results of the present proof-of-concept trial suggest that a single session of left DLPFC-targeted tDCS does not influence psychophysiological responses during exercise in the heavy intensity domain in healthy individuals. In addition, tDCS did not affect brain oxygenation at rest or during vigorous exercise. Future studies should consider evaluating other population samples that might be more prone to presenting negative psychophysiological responses (e.g., individuals with mental health disorders).

## Data Availability

The raw data supporting the conclusions of this article will be made available by the authors upon reasonable request.
